# Abdominal Wall Sinus and Recurrent Abscess Formation Due to a Foreign Body Following Surgical Intervention

**DOI:** 10.7759/cureus.49013

**Published:** 2023-11-18

**Authors:** Edilson Araujo Sabino, Guilherme Pasquini Cavassin, Juliano Xavier Santos, João Arthur Borges da Silva, Henrique Waldraff Teixeira

**Affiliations:** 1 General Surgery, Complexo Hospitalar do Trabalhador, Curitiba, BRA; 2 Medicine, Universidade Federal do Paraná, Curitiba, BRA; 3 Medicine, Universidade Positivo, Curitiba, BRA

**Keywords:** multidisciplinary approach, foreign body reaction, surgical complications, recurrent abscesses, sinus formation

## Abstract

The sinus tract, a tubular structure with no outlet connecting deep tissues to the skin, is a rare entity, especially in patients undergoing abdominal surgeries. The pathophysiology involves factors such as liquefaction of adipose tissue, infection, and retention of foreign bodies. Inadequate surgical drainage can lead to chronicity, culminating in the formation of an infectious sinus in the abdominal wall, clinically known as a sinus. Understanding this phenomenon is crucial to avoid recurrences and complications. A 65-year-old female patient with a history of multiple abdominal surgeries presented with pain and suprapubic discharge. Similar episodes had occurred previously. Examinations revealed a fistulous tract in the right iliac fossa. The surgical intervention included the excision of the tract, identification of points with cotton thread, and antibiotic therapy. Follow-up in the outpatient setting showed no recurrences. The presence of postoperative foreign bodies, such as sutures, can trigger recurrent local infections. Diagnosis involves imaging studies, and the type of surgical thread influences complications. Treatment aims at drainage and excision of the tract. A multidisciplinary approach is of paramount importance. The sinus, with its insidious formation, highlights the complexity of this condition. Careful selection of surgical materials, precise imaging diagnosis, and a multidisciplinary approach are essential for effective treatment. This case emphasizes the importance of clinical practice to enhance clinical outcomes and the quality of life of patients affected by this challenging condition.

## Introduction

The sinus tract, a blind-ended tubular structure that connects deeper tissues to the skin, represents a rare nosological entity, with a higher incidence in patients who have undergone abdominal surgical interventions, especially in cases of infected surgeries, traumas, and damage control procedures [1,2]. The underlying pathophysiology of this complex condition can be triggered by various factors, such as liquefaction of adipose tissue, pyogenic infection, or retention of foreign bodies, such as implants and surgical threads [1-3].

Inadequate drainage of the tissue resulting from the inflammatory process through the surgical incision substantially increases the risk of chronicity of the condition. This pathological evolution culminates in the progressive formation of granulation tissue, followed by the constitution of scar tissue. Gradually, this process results in the formation of an infectious sinus in the abdominal wall, clinically defined as a sinus [4-6]. Understanding the intricate network of events leading to sinus formation is crucial for effective approaches, as neglecting it can result in persistent recurrences and significant clinical complications.

## Case presentation

Clinical history

A 65-year-old female patient sought emergency care with localized pain in the hypogastric transition and right iliac fossa. The pain, insidious and progressive in onset, was accompanied by edema, cutaneous hyperemia, and the discharge of secretion through an orifice in the suprapubic region. The patient reported similar symptoms in previous episodes, specifically in 2014, 2018, and 2019, when she sought medical attention at another facility and underwent percutaneous drainage procedures. The surgical history revealed three cesarean sections (the last one in 1985), a hysterectomy with a median infraumbilical incision in 1987, and an open appendectomy with a McBurney incision in 1996.

Physical examination

**Figure 1:**
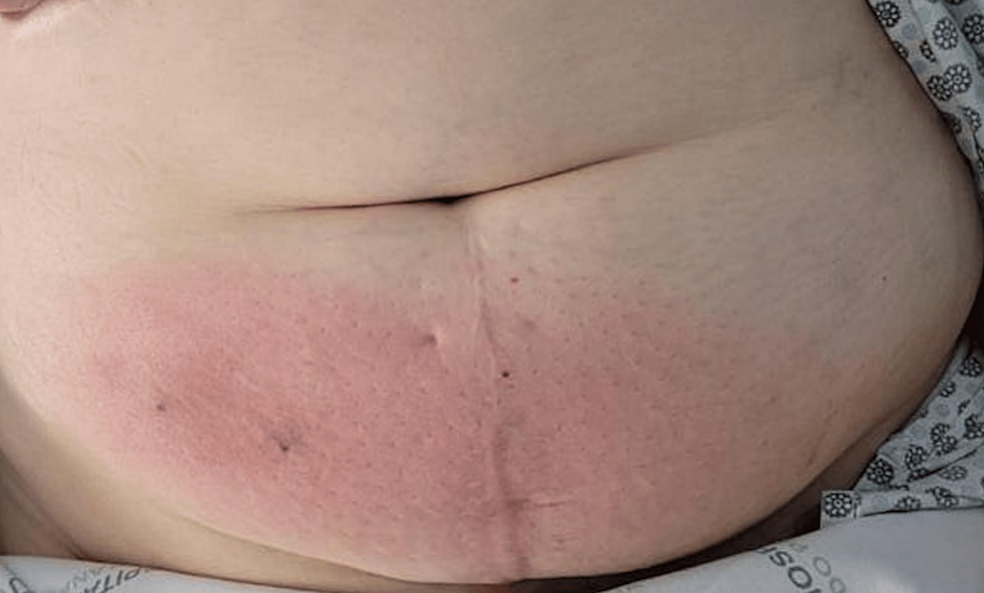
Lower abdomen with inflammatory signs at admission. The purulent fluid was drained through a hole beneath the patient’s abdominal fold.

During the abdominal inspection, inflammatory signs were identified in the lower right abdomen, with a notable seropurulent drainage orifice located 3 cm from the pubic symphysis. Upon palpation, a localized area of induration was observed, with no evidence of peritoneal signs (Figure 1).

Diagnostic investigation

**Figure 2:**
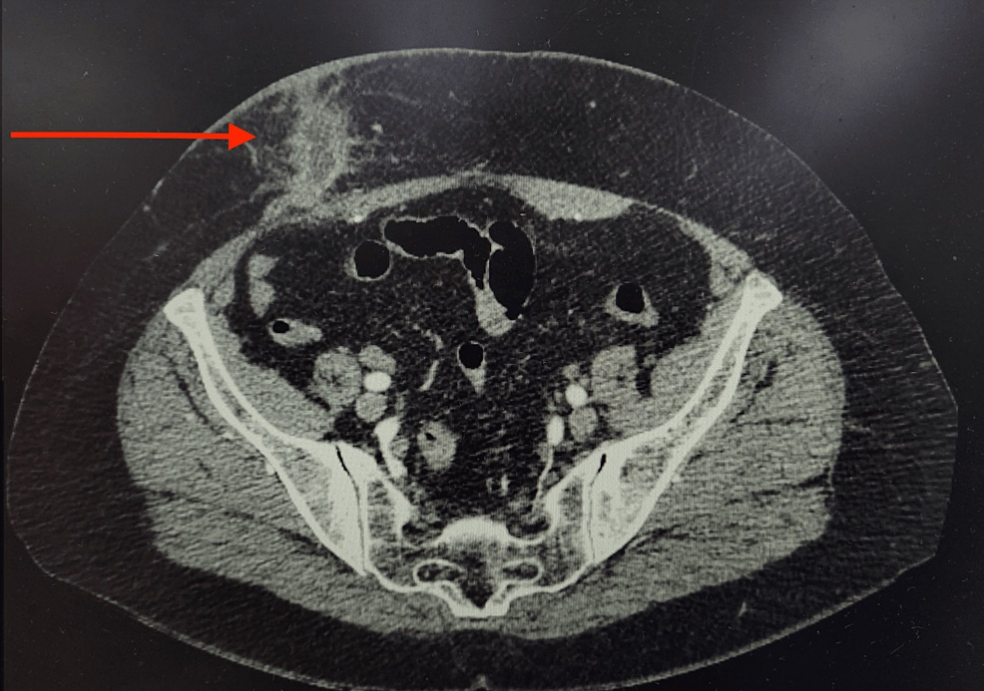
CT abdomen: an area of subcutaneous fat densification and liquefaction with the fistulous tract.

When faced with the clinical scenario, the decision was made to admit the patient for a more comprehensive evaluation. Abdominal computed tomography (CT) was recommended for precise surgical planning, as previous attempts at percutaneous drainage in other facilities had proven ineffective. The CT scan revealed an area of densification and liquefaction of subcutaneous fat in the right iliac fossa, accompanied by an extraperitoneal fistulous tract draining into the suprapubic region, measuring approximately 12 cm (Figure 2).

Surgical intervention

**Figure 3:**
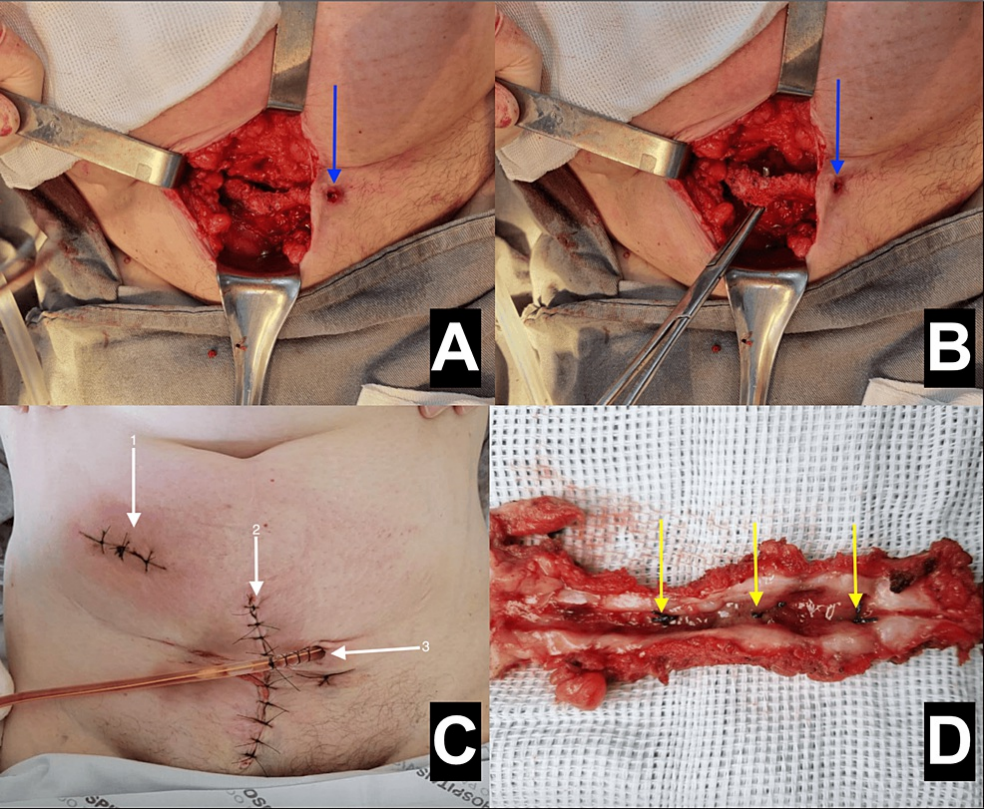
(A,B) Resection of the fistula proximally and distally. (C) Postoperative result and vacuum drain inserted. (D) Identification of points with non-absorbable cotton thread in the fistulous tract. (A,B) Blue arrows are pointing to the exit hole of the drainage. (C) Arrow 1 is pointing to the McBurney point incision for proximal fistula resection. Arrow 2 is pointing to the infraumbilical median incision for distal resection of the fistula, where the purulent drainage was coming from. Arrow 3 is pointing to the vacuum drain inserted. (D) Yellow arrows are pointing to the cotton thread in the subcutaneous tissue.

The patient was taken to the operating room, where a moderate amount of purulent secretion was drained. The fistula was excised, both proximally in the McBurney region and distally in the mid-infraumbilical region (Figures 3A, 3B). Subcutaneous approximation was done using 2.0 Vicryl, and a vacuum drain was inserted (Figure 3C). During the opening of the fistulous tract after excision, points made with non-absorbable thread (cotton) were identified (Figure 3D).

Postoperative and follow-up

During hospitalization, the patient showed a significant improvement in pain and abdominal inflammatory signs. In the subsequent days, minimal serosanguinous drainage was observed from the vacuum drain. Hospital discharge was granted on the seventh postoperative day, with antibiotic therapy guided by culture results (positive for methicillin-resistant *Staphylococcus aureus* (MRSA)). Ambulatory follow-up was scheduled for continuous monitoring. The histopathological examination confirmed the suspicions, revealing fibro-adipose and muscular tissue with fibrosis consistent with the fistulous tract and sinus.

**Figure 4:**
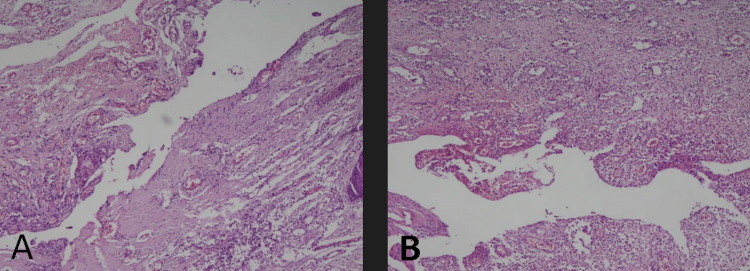
(A,B) Optical microscopy at 100× magnification. Hematoxylin and eosin staining. Areas of discontinuity delimited by fibro-adipose granulation tissue and muscular fibrosis, moderate lymphoplasmacytic inflammatory infiltrate, and fatty necrosis, consistent with a fistulous tract.

## Discussion

Postoperative retention of various foreign bodies, such as gallstones, gauze, food residues, and, as evidenced in this case, cotton suture threads, can trigger a prolonged immune response, resulting in the recurrence of local infections that clinically manifest after long periods, often years [4,7]. In the presented context, the use of cotton thread in the subcutaneous tissue emerged as the causative agent, emphasizing the importance of careful selection of surgical materials to prevent late complications.

As well in some cases of fistulas, the sinus tract demonstrates a chronic nature, characterized by persistent pain, cutaneous hyperemia and edema, and drainage of serous or purulent content [8,9]. Laboratory tests play a crucial role in suggesting an infectious condition, with common findings of leukocytosis and elevated inflammatory markers [8]. Clinical suspicion becomes relevant in patients with persistent drainage, even under antibiotic therapy and percutaneous drainage. However, it is imperative to consider other differential diagnoses, such as tuberculosis and malignancies [10].

The choice of surgical thread type proves to be fundamental in preventing complications. Absorbable and/or monofilament threads demonstrate a lower risk of adhesion or abscess formation [11]. A comprehensive systematic review involving 15 studies and a significant sample of 5,875 patients highlights the lower occurrence of fistula or sinus formation when polyglactin threads are used, proving superior to PDS, polypropylene, and nylon threads [12].

Regarding the diagnosis, the difficulty in clinically predicting the size, depth, and structures involved in the fistula or sinus emphasizes the importance of imaging examinations for effective surgical planning. Ultrasonography (USG) stands out for real-time visualization of superficial lesions, while CT with 3D reconstruction and magnetic resonance imaging (MRI) provide detailed information about the lesions and their relationships with surrounding structures [2,12]. Although contrasted imaging is effective in tracing the path, its limitation in visualizing anatomical relationships of adjacent tissues needs to be considered.

The treatment of the sinus tract is based on draining the infectious content and resecting the tract. While endoscopic therapies are being studied to reduce costs and morbidity associated with treatment, their effectiveness still lacks conclusive evidence [13]. The decision on the need for antibiotic therapy should be individualized, considering factors such as positive microbiological culture, bacterial resistance, and the patient’s clinical response. A multidisciplinary approach with surgeons, infectious disease specialists, and radiologists is essential to ensure a comprehensive and effective treatment.

The sinus, with its insidious formation and gradual clinical manifestation over months to years, highlights the complexity and persistence of this nosological entity. Its intricate etiology results from an immune response triggered by the presence of foreign bodies, often associated with infectious processes. A comprehensive understanding of this phenomenon requires a multidisciplinary approach that integrates the expertise of surgeons, infectious disease specialists, and radiologists.

The relevance of imaging in surgical planning cannot be underestimated, as it allows for detailed visualization of the fistulous tract, assisting in determining the extent and depth of the lesion. Tools such as CT with 3D reconstruction, MRI, and USG emerge as fundamental pillars in this diagnostic process.

The treatment of the sinus is based on the fundamental principles of draining the infectious content and resecting the fistulous tract. The careful choice of surgical materials, especially absorbable and monofilament threads, represents an effective preventive strategy against complications such as adhesions and abscesses. While endoscopic therapies are under investigation to optimize costs and reduce morbidity, their effectiveness still lacks conclusive validation.

## Conclusions

In summary, this case report emphasizes the importance of an integrated and personalized approach for patients presenting with a sinus, from diagnostic investigation to the implementation of therapeutic strategies. The continuous search for innovations in clinical and surgical practices is essential to enhance clinical outcomes and the quality of life for patients affected by this condition.
